# Partner-dependent communication without dynamic adaptation in autism

**DOI:** 10.1177/13623613251410418

**Published:** 2026-01-31

**Authors:** Saskia BJ Koch, Jordy van Langen, Jana Bašnáková, Arjen Stolk

**Affiliations:** 1 Donders Institute for Brain, Cognition and Behaviour, Center for Cognitive Neuroimaging, Radboud University, Nijmegen, The Netherlands; 2 Behavioral Science Institute, Radboud University, Nijmegen, the Netherlands; 3 Donders Institute for Brain, Cognition and Behaviour, Medical Neuroscience Department, Radboud University Medical Center, The Netherlands; 4 Institute of Experimental Psychology, Centre of Social and Psychological Sciences SAS, Slovakia; 5 Psychological and Brain Sciences, Dartmouth College, USA

**Keywords:** audience design, social anxiety, social development, social interaction, stereotypes

## Abstract

Autism is a neurodevelopmental condition characterized by persistent communication challenges, often compounded by social anxiety. Although differences in perspective-taking, cognitive flexibility, and social motivation have been implicated in these challenges, their influence on live interpersonal interactions remains unclear. In this study, we quantitatively examined how autistic and non-autistic individuals with varying levels of social anxiety adapted their communication during experimentally controlled interactions with two ostensibly distinct partners—a child and an adult—both portrayed by the same role-blind confederate. Autistic participants were equally motivated and capable as non-autistic participants in adjusting their communication to stereotypical assumptions about a partner’s abilities, spontaneously using greater emphasis when addressing the presumed less capable child. However, they were less likely to modify these stereotype-driven behaviors in response to interaction-based evidence of partners’ equal competence. While non-autistic participants dynamically adapted their communication to treat both partners equivalently, autistic participants maintained their stereotype-driven adjustments throughout the interaction. Preregistered analyses further linked non-autistic individuals’ adaptive responses to early social exposure, a developmental factor not observed in autistic participants. Together, these findings highlight a core interactional capacity, shaped by early social experiences and operating on interaction-based evidence, as central to understanding communication challenges in autism.

**Lay abstract:**

Everyday communication can be challenging for autistic individuals, particularly when social anxiety is involved. Research suggests that differences in understanding and adapting to others may contribute to these challenges, but it remains unclear whether and how these differences affect real-time interactions. This study invited autistic and non-autistic participants with varying levels of social anxiety to interact online with two “partners.” One partner was introduced as a child and the other as an adult, although in reality the same actor played both roles without knowing which role he was assigned. All participants initially emphasized their communication more with the presumed child, whom they assumed was less capable. Over time, however, non-autistic participants adapted their approach, treating both partners equally as they gathered evidence that the child was just as competent as the adult. In contrast, autistic participants continued to treat the child as less capable throughout the interaction. Moreover, non-autistic participants who adapted more quickly tended to have experienced greater early social exposure in daycare, a relationship that was not observed in autistic participants. These findings suggest that while autistic individuals are willing and able to adjust their communication based on initial assumptions about others, they are less likely to revise these adjustments in response to evidence of a partner’s actual understanding during interaction, a skill that appears to develop differently for them from an early age.

## Introduction

Communication challenges are a defining feature of autism, profoundly shaping social interactions and daily life (American Psychiatric Association 2013). These difficulties are frequently compounded by social anxiety, which affects up to 50% of autistic individuals compared with about 10% of the general population (Maddox & White, 2015; Spain et al., 2018). Proposed contributors include differences in perspective-taking, cognitive flexibility, and intrinsic motivation for social engagement (Baron-Cohen et al., 1985; Chevallier et al., 2012; Geurts et al., 2009). Yet little is known about how these factors shape communication during live interpersonal exchanges, where social anxiety may also prevail. The present study addresses this gap by examining how autistic and non-autistic individuals with high or low social anxiety adapt their communication during precisely quantified interactions with different partners.

At the heart of interpersonal communication lies the ability to tailor one’s message to what a partner is likely to understand (Clark & Murphy, 1982). For instance, adults often simplify their language and exaggerate intonation when addressing children, operating on the assumption that such modifications aid comprehension for younger and presumed less capable listeners (Csibra & Gergely, 2011; Snow, 1972). Such adjustments reflect underlying expectations about social categories, which can be understood as stereotypes: generalized beliefs that provide quick, though not always accurate, predictions about individuals (Hilton & von Hippel, 1996). It has been argued that autistic individuals may rely less on these stereotype-based assumptions, potentially contributing to atypical prosody and other communicative differences observed in social settings (Cho et al., 2019; Fusaroli et al., 2017; Kirchner et al., 2012; Zalla et al., 2014). However, in tasks that explicitly require applying stereotypes, such as judging competence or trustworthiness from photographs, autistic individuals perform comparably to non-autistic peers (Da Fonseca et al., 2011; White et al., 2006). Whether autistic individuals spontaneously draw on stereotypes during live interpersonal interactions, where such assumptions must be applied flexibly and selectively, remains an open question.

Beyond stereotype-driven assumptions, effective communication also depends on integrating evidence arising from the interaction itself (Clark, 1996; Koch et al., 2024). Communicators must adapt, for example, if a presumed novice partner demonstrates unexpected proficiency (Kuhlen & Brennan, 2010). Such adaptability requires monitoring both explicit signals of understanding (e.g. affirmations like “uh-huh” or “I see”) and more implicit cues, including sustained eye contact or the smooth continuation of conversation with a relevant next utterance (Brennan et al., 2010). While autistic individuals often incorporate explicit verbal signals (Slocombe et al., 2013), their responsiveness to implicit cues of comprehension or engagement appears reduced (Cola et al., 2022; Du Bois et al., 2014; Rifai et al., 2022; Tager-Flusberg & Anderson, 1991). These observations raise the possibility that communication challenges in autism may arise less from an unwillingness to adapt and more from difficulties in integrating interaction-based evidence.

Here, we quantitatively examine how autistic and non-autistic individuals integrate stereotype-driven assumptions and interaction-based evidence in an experimentally controlled communication game. Participants alternately interacted with two presumed partners—a child and an adult—on a digital game board, guiding them to a hidden object. The computer-mediated setup, which excluded direct verbal or face-to-face contact, enabled precise measurement of communicative dynamics while minimizing the influence of linguistic conventions (Galantucci & Garrod, 2011). To isolate the effects of stereotypes, both partner roles were performed by the same role-blind confederate, ensuring that any behavioral differences reflected participants’ beliefs about partner ability rather than actual performance. This design created a systematic tension between stereotype-based expectations, which framed one partner as a less capable child, and accumulating interaction-based evidence demonstrating that both partners were equally competent. Stereotype-driven adjustments were indexed by participants’ increased emphasis when communicating with the presumed child, whereas interaction-driven adjustments were measured by the extent to which communicative behavior converged toward the partners’ matched performance. We further investigated whether group differences in interaction-driven adjustments stemmed from reduced responsiveness to explicit feedback provided after each trial or diminished sensitivity to implicit cues of equal competence embedded in the confederate’s consistent behavior.

Finally, our study builds on developmental research showing that early social exposure in daycare has lasting effects on interaction-driven adjustments into adolescence, independent of family background or later social environments (Koch et al., 2024). Coinciding with a critical developmental period, daycare may foster adaptability by exposing individuals to diverse communicative challenges, such as interacting with peers from varied backgrounds and forming affiliative bonds with non-familial caregivers (Chapin, 1915; Hrdy, 2009; Liberman et al., 2017). If autistic individuals derive less benefit from such early exposure (Fasano et al., 2021), despite retaining the ability to acquire and apply stereotypes (Hirschfeld et al., 2007), this could give rise to a phenotype characterized by preserved stereotype-based but reduced interaction-driven adjustments, independent of social motivation or anxiety. To test this possibility, we examined whether the developmental benefits of early social exposure extend into adulthood, and whether they influence interaction-driven adjustments in autistic individuals to the same degree as in non-autistic peers.

## Method

### Participants

The study enrolled 144 participants, divided into three groups: autistic adults (*n* = 48), non-autistic peers with low social anxiety (low SA, *n* = 48), and non-autistic peers with high social anxiety (high SA, *n* = 48). All participants were drawn from an established pool of well-characterized individuals who had previously completed psychometric assessments as part of neuroimaging research (Mangnus et al., 2024). Autistic participants had originally been recruited through social media, outpatient clinics, and a clinical organization supporting students with autism. They met diagnostic criteria for Autism Spectrum Disorder as determined by a qualified clinician, and their diagnostic status was confirmed via telephone screening. Non-autistic participants had originally been recruited via social media and the Radboud University participant database, and were assigned to the low-SA (LSAS < 30) or high-SA (LSAS ⩾ 30) groups based on their scores on the Liebowitz Social Anxiety Scale (Liebowitz, 1987; Mennin et al., 2002; Oakman et al., 2003).

**Figure 1.**
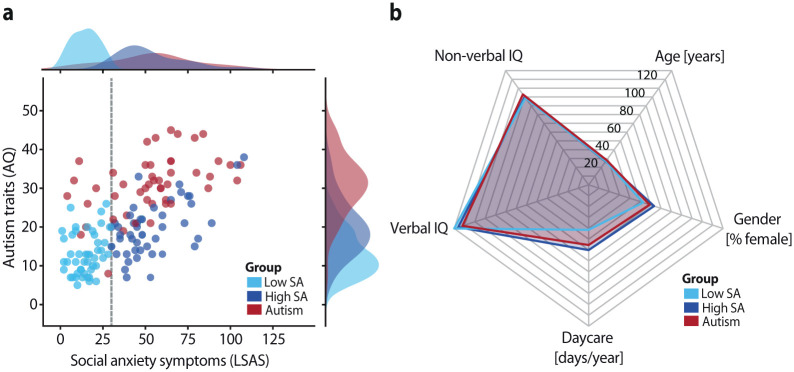
Clinical symptom distribution and demographics: (a) Distribution of autistic traits (Autism-Spectrum Quotient, AQ) and social anxiety symptoms (Liebowitz Social Anxiety Scale, LSAS) across participant groups. A cutoff score of 30 on the LSAS (dashed line) was used to classify non-autistic participants into low and high social anxiety (SA) groups. (b) Participant groups were matched on age, sex, verbal IQ (Wechsler Adult Intelligence Scale; Wechsler, 1997), nonverbal IQ (Raven’s Progressive Matrices; Raven, 1989), and daycare attendance. For visualization, daycare attendance is displayed as the total number of days per year.

Autistic participants scored significantly higher on the Autism-Spectrum Quotient (AQ; Baron-Cohen et al., 2001) than both comparison groups (low SA: *t*_(93)_ = 13.21, *p* < .001, Cohen’s *d* = 2.71; high SA: *t_(_*_94)_ = 7.63, *p* < .001, Cohen’s *d* = 1.56), and high-SA participants reported more autistic traits than low-SA participants (*t*_(93)_ = −5.12, *p* < .001, Cohen’s *d* = 1.05). Social anxiety levels were comparable between the autism and high-SA groups (*t*_(94)_ = 0.09, *p* = .927, *BF_01_* = 4.64), and both reported significantly higher social anxiety than the low-SA group (autism: *t*_(94)_ = 10.24, *p* < .001, Cohen’s *d* = 2.09; high SA: *t*_(94)_ = 13.14, *p* *<* .001, Cohen’s *d* = 2.68; Figure 1(a)). The groups were matched on age, biological sex, verbal and nonverbal IQ, and daycare attendance, as confirmed by frequentist and Bayesian analyses (all *p* > .05; all *BF_01_* > 4; see Figure 1(b) and Supplemental Table S1). These results confirmed the expected group distinctions, enabling the isolation of communicative alterations in the autism group independent of social anxiety and demographic factors.

Five participants were excluded for failing to understand or accept the partner manipulation (Supplemental Results), resulting in a final sample of 46 autism, 45 low-SA, and 48 high-SA participants. The study was approved by the local ethics committee (Committee on Research Involving Human Subjects, Arnhem-Nijmegen region, the Netherlands, file number 2019-6059) and conducted in accordance with the Declaration of Helsinki. All participants provided written informed consent and were financially compensated for their time and travel expenses.

### Task

**Figure 2.**
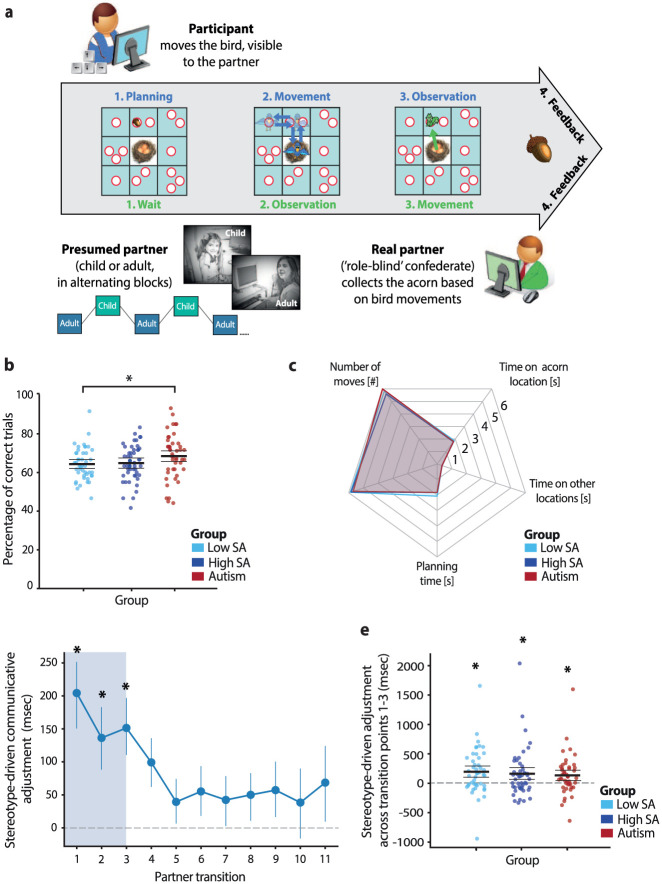
Task performance and stereotype-driven adjustments: (a) Overview of the communication game. In Phase 1, participants were shown the acorn’s location on a digital game board. In Phase 2, they moved a bird avatar to indicate the location to their partner. In Phase 3, the partner, represented by a squirrel avatar, selected the location based on the bird’s movement. Feedback was provided in Phase 4, with successful trials marked by a large acorn and unsuccessful trials by a crossed-out acorn. Participants alternated between presumed child and adult partners, with both roles performed by a role-blind confederate to ensure consistent performance. (b) Overall task performance was comparable across groups, with autistic participants performing on par with or slightly better than non-autistic peers, regardless of social anxiety (SA) level. (c) General task measures such as planning time, movement time, number of moves, and time spent on acorn and non-acorn locations did not differ significantly across groups. (d) Participants spent more time on the acorn’s square when interacting with the presumed child. This stereotype-driven adjustment was most pronounced during the early stages of interaction (partner transitions 1–3) and declined over time. (e) All groups showed this initial adjustment (averaged across the first three partner transitions), with no significant group differences. Untransformed values are displayed in milliseconds. Error bars represent the standard error of the mean; * *p* < .05.

Participants engaged in a communication game adapted from previous studies and modified for remote administration (de Boer et al., 2017; Koch et al., 2024; Newman-Norlund et al., 2009; Stolk et al., 2013, 2015). Each participant was paired with a confederate experimenter, and the dyad played the game online from separate locations with no direct verbal or face-to-face contact. The interaction took place entirely on a 3 × 3 grid displayed simultaneously on both players’ screens. Across 60 trials, participants guided their partner (the confederate) to collect an acorn hidden in one of 15 possible locations, represented by white circles on the grid (Figure 2(a)). The acorn’s location was revealed only to the participant at the start of each trial (Phase 1). To communicate this location, participants moved a bird avatar across the grid using keyboard arrow keys (Phase 2). The bird’s movements, visible to both players, were restricted to horizontal and vertical paths between the centers of the nine squares. Participants had 10 s to complete their movements and return the bird to the grid’s center. Once the bird returned, or when the time limit expired, a squirrel avatar appeared at the grid’s center, signaling the partner’s turn (Phase 3). The confederate then moved the squirrel freely to the circle inferred from the bird’s movements and selected it with a mouse click. Feedback followed immediately after the selection (Phase 4). Successful trials, in which the partner correctly identified the acorn’s location and the participant returned the bird in time, were marked by a large acorn, whereas unsuccessful trials were indicated by a small acorn crossed in red.

Task difficulty was manipulated by varying the spatial relationship between the bird’s allowable positions and the acorn’s possible locations, with difficulty progressively increasing across trials in a pseudo-randomized order. When a square contained multiple circles, the bird could not be unambiguously linked to the acorn’s location, requiring dyads to negotiate a shared strategy from an open-ended set of possibilities. Because no predefined solutions existed and the squirrel could not simply mimic the bird’s path, dyads had to establish and coordinate on communicatively relevant aspects of the bird’s movements as the interaction unfolded. Strategies typically range from using entry or exit directions, to briefly stepping out of and back into the intended square (see Figure 2(a) for an example), to creating more elaborate drawings across the grid (de Boer et al., 2017). Even in trials with only a single circle per square, participants still needed to disambiguate the intended square from others traversed by the bird, often by spending additional time or emphasis on it.

### Experimental design

Participants accessed the game via a web server and played it from home or another distraction-free environment. After reading the task instructions, they completed three practice trials to familiarize themselves with the game mechanics, using the bird avatar to indicate the acorn’s location and the squirrel avatar to collect it. Once practice was complete, the confederate experimenter briefly called the participant to confirm their understanding of the instructions and to explain that the game would involve alternating between two online partners: an adult and a 5-year-old (Figure 2(a)). After this call, the confederate hung up and proceeded to perform both partner roles while remaining blind to the acorn’s location, role assignment, and the participant’s group status (autism, low SA, or high SA).

The experiment consisted of 12 blocks of five trials each, alternating between the presumed child and adult partners. Before each block, a photograph of the presumed partner was displayed in full screen and then remained visible in the top-right corner of the participant’s screen throughout the block. The order of partner alternations and goal configurations was counterbalanced across participants using a random seed (partner order: *χ*^2^_(2)_ = 1.56, *p* = .458, *BF_01_* = 8.56; goal sets: *χ*^2^_(2)_ = 0.22, *p* = .895, *BF_01_* = 16.53). The game was implemented using *nodeGame* (Balietti, 2017) and lasted an average of 13 ± 2 min. After completing the game, participants filled out questionnaires assessing early-life social experiences and their beliefs about the cognitive abilities of their interaction partners, providing a check on the effectiveness of the partner manipulation (de Boer et al., 2017; Newman-Norlund et al., 2009).

### Data analysis

Analyses were preregistered and focused on three primary outcomes: (1) stereotype-driven adjustments, (2) interaction-based adjustments, and (3) the influence of early social exposure (Koch et al., 2022). To assess the specificity of these effects, we also tested whether group differences in adjustments were confined to communicatively relevant areas of the game board rather than reflecting general task behaviors. General behaviors included planning time (Phase 1, Figure 2(a)), movement time, number of moves, and the average time spent on the acorn’s location or other grid areas (Phase 2). In addition, we examined participants’ communicative strategies to evaluate whether autistic and non-autistic groups showed comparable strategy use and modification.

All continuous measures were log-transformed to meet normality assumptions and analyzed using repeated-measures analyses of variance (ANOVAs), with group (autism, low SA, high SA) as a between-subjects factor and partner (child, adult) as a within-subjects factor. Analyses were conducted in R using the *rstatix* package (Kassambara, 2023). Additional checks confirmed that observed adjustments reflected participants’ beliefs about their partners’ cognitive abilities rather than differences in confederate performance (Phase 3). Communicative success (Phase 4) was analyzed at the trial level using a Bayesian linear mixed-effects model in R with the *brms* package (Bürkner, 2017). The dependent variable was accuracy (0 = incorrect, 1 = correct), with group as a between-subjects factor and task difficulty as a covariate. Random intercepts were included for participants. Bayesian models were estimated with two parallel chains of 2,000 iterations each (1,000 warm-up, 1,000 for posterior inference), ensuring convergence and stability. Effects were considered reliable if the 95% credible interval (CI) did not include zero, and posterior probabilities (*pp*), analogous to *p*-values, were reported as a measure of evidence (Makowski et al., 2019).

### Stereotype-driven adjustments

Previous studies using this task consistently show that participants spend more time with the bird in the acorn’s square when interacting with a presumed younger partner (de Boer et al., 2017; Koch et al., 2024; Newman-Norlund et al., 2009; Stolk et al., 2013, 2015). This behavior parallels the prosodic modifications often observed in child-directed speech (Grieser & Kuhl, 1988). The present study leveraged these timing adjustments as a quantitative marker of participants’ stereotype-based beliefs about their partner, measured in milliseconds.

To capture the temporal dynamics of these adjustments, time spent on the acorn’s location was residualized against planning time and task difficulty using a linear mixed-effects model implemented in R with the *lme4* package (Bates et al., 2015). Task difficulty was categorized into three levels: (1) acorn in a square with one circle, (2) acorn in a square with multiple circles that could be disambiguated by stepping in and out, and (3) acorn near the border of the game board in a square with multiple circles that could not be disambiguated by stepping in and out. Trials with missing data (2.45%) were excluded. Adjustment estimates were computed as block-by-block differences in time spent on the acorn’s location for each of the 11 partner transitions (Liu et al., 2021). These values were multiplied by the expected direction of the effect (+1 for adult-to-child transitions, −1 for child-to-adult transitions), such that positive values indicated adjustments toward the child partner (Figure 2(a)).

Significant temporal clusters of adjustments were identified using cluster-based permutation testing with threshold-free cluster enhancement (TFCE) applied to one-sample *t*-tests, with 10,000 Monte Carlo permutations and a significance threshold of *p* < .05 (Maris & Oostenveld, 2007). Average adjustments within significant clusters were then compared across groups using a one-way ANOVA, followed by one-sample *t*-tests per group with a Bonferroni-corrected significance threshold of *p* < .017 (.05/3 tests). Finally, robustness was confirmed by repeating the analyses after excluding one high SA outlier.

### Interaction-driven adjustments

Interaction-driven adjustments reflect the gradual decline in stereotype-driven adjustments over time as participants accumulated interaction-based evidence against the presumption of differing communicative understanding between the two partners. This convergence was modeled as a linear effect of partner transitions (1 to 11) on stereotype-driven adjustments using a Bayesian linear mixed-effects model in R with the *brms* package (Bürkner, 2017). The dependent variable was stereotype-driven adjustment, with group (autism, low SA, high SA) and daycare attendance (see section “Socio-developmental factors”) as between-subjects factors, and partner transition point as a within-subjects factor. Participant was included as a random effect to account for individual differences, and a Student’s *t*-distribution was specified to reduce sensitivity to outliers.

Follow-up analyses tested the linear decrease in stereotype-driven adjustments within each group separately, applying a corrected threshold of *pp* < .017 (.05/3 tests). Robustness was further confirmed by repeating the analyses after excluding three outliers (two low-SA participants and one high-SA participant).

### Socio-developmental factors

Early social exposure was quantified as the average number of days per week spent in daycare or with childminders between ages 0 and 4 (*M*: 1.15 ± 1.00, range = 0–5), excluding care provided by family members. In the Netherlands, children typically begin elementary school at age 4. Consistent with prior work (Koch et al., 2024; Stolk et al., 2013), familial factors were also assessed, including socioeconomic status (indexed by parents’ average education level on a 10-point scale; *M* = 6.09 ± 2.04, range = 1–10) and number of siblings (*M* = 1.39 ± 0.92, range = 0–5). To examine whether early social exposure predicted adaptability, participants’ rates of convergence were estimated as the linear slope across the 11 partner transitions using MATLAB’s *robustfit* function. Partial Spearman rank correlations then tested the association between daycare attendance and convergence rates within the autism and comparison groups, controlling for intercept effects (slope-intercept correlation: *ρ*_(139)_ = −.881, *p* < .001). One-sided tests were used for preregistered hypotheses with directional predictions based on prior work; all other correlations were evaluated two-sided.

Additional analyses confirmed that the effects of early social exposure remained significant after accounting for familial factors. To enhance statistical power, comparison group data were pooled, and effect sizes were verified to be consistent with previous findings in a larger developmental sample (Koch et al., 2024). To rule out concerns of underpowering in the autism group, we tested for group differences in the magnitude of the correlation coefficients. In addition, data from both groups were combined, and the observed effects were examined for significant attenuation in the combined sample relative to the comparison group alone.

## Results

### Task performance

Participants successfully guided their partners to the hidden acorn locations with an average accuracy of 65.88 ± 9.90%, well above chance level (6.67%, 1/15 possible locations). As shown in Figure 2(b), autistic participants performed slightly better (68.45 ± 11.49%) than the low-SA group (64.36 ± 8.40%; *B* = −0.32, 95% CI = [−0.58, −0.07], *pp* = .018) and showed a marginal trend toward higher performance relative to the high-SA group (64.84% ± 9.26%; *B* = −0.24, 95% CI = [−0.50, 0.01], *pp* = .056). No significant group differences were observed in general task behaviors, including planning time, movement time, number of moves, or time spent on the acorn’s location or other areas of the game board (all *F* ⩽ .163, all *p* ⩾ .199; Figure 2(c)), nor in the variety or distribution of communicative strategies (Supplemental Results). Communicative success did not differ significantly between presumed child and adult partners (*B* = −0.12, 95% CI = [−0.30, 0.06], *pp* = .192), and there were no significant group-by-partner interaction effects (all *B* ⩽ 0.16, all *pp* ⩾ .20). Overall, autistic participants performed on par with, and in some cases slightly better than, non-autistic peers, with communicative success consistent across partners.

### Stereotype-driven adjustments

Participants spent more time on the acorn’s location when interacting with the presumed child partner (*M difference* = 94.48 ms out of ~1,850 ms total, *F*_(1,136)_ = 24.02, *p* < .001; Supplemental Figure S1). This adjustment was specific to communicatively relevant areas of the board and did not extend to other task behaviors (Supplemental Results). These subtle timing differences, largely unnoticed by participants, were driven by stereotypical beliefs about their partners’ abilities, as confirmed by the confederate’s consistent performance across roles and post-task questionnaire responses (Supplemental Results). Adjustments were strongest early in the interaction (partner transitions 1–3) and declined over time (Figure 2(d)). No significant group differences emerged in these initial adjustments (*F*_(2,136)_ = 0.09, *p* = .912, *BF*_01_ = 13.17). However, significant effects were observed within each group (low SA: *t*_(44)_ = 2.93, *p* = .005, Cohen’s *d* = 0.44; high SA: *t*_(47)_ = 2.81, *p* = .007, Cohen’s *d* = 0.40; autism: *t*_(45)_ = 3.08, *p* = .004, Cohen’s *d* = 0.45; Figure 2(e)). Thus, autistic participants, like their non-autistic peers, spontaneously modulated their communicative behavior in line with presumed partner abilities.

### Interaction-driven adjustments

**Figure 3.**
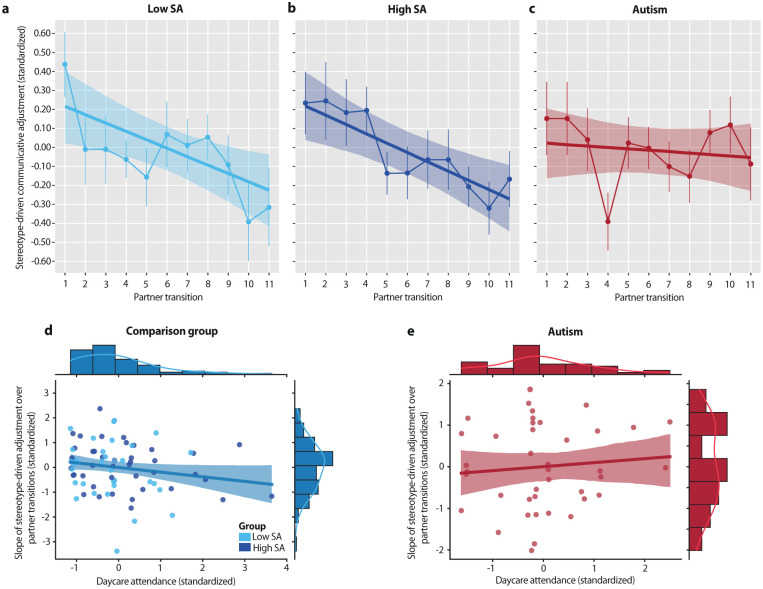
Interaction-driven adjustments and modulation by early social exposure: (a, b) Low and high social anxiety (SA) participants showed a linear decline in stereotype-driven adjustments across partner transitions, illustrated by model slope estimates with overlaid adjustment values. Error bars indicate the standard error of the mean. (c) In contrast, autistic participants showed no evidence of decline, maintaining their initial stereotype-driven adjustments across transitions. (d) For low- and high-SA participants, greater time spent in daycare during early childhood was associated with a steeper decline in stereotype-driven adjustments. (e) For autistic individuals, no significant relationship was observed between daycare attendance and these interaction-driven adjustments. Scatterplots display standardized residuals corrected for the intercept of the linear fit.

As participants accumulated evidence against presumed partner differences, stereotype-driven adjustments generally declined across the 11 partner transitions. Autistic individuals, however, showed a smaller decrease compared to the high-SA group (*B* = −0.04, 95% CI = [−0.08, −0.00], *pp* = .038) and a similar trend relative to the low-SA group (*B* = −0.04, 95% CI = [−0.08, 0.00], *pp* = .052). Both low- and high-SA groups exhibited significant linear decreases (low SA: *B* = −0.04, 95% CI = [−0.07, −0.01], *pp* = .005; high SA: *B* = −0.05, 95% CI = [−0.08, −0.02], *pp* < .001; Figure 3(a) and (b)), whereas autistic participants showed no reliable change (*B* = −0.01, 95% CI = [−0.04, 0.02], *pp* = .604; Figure 3(c)). These results suggest that autistic participants were less influenced by accumulating interaction-based evidence, maintaining their initial child-directed adjustments throughout the interaction.

### Responsiveness to performance feedback

Across all groups, participants increased the time spent on the acorn’s square after receiving negative feedback (a crossed-out acorn during Phase 4; *t*_(138)_ = 2.70, *p* = .008, Cohen’s *d* = 0.229). No significant group differences were observed in this post-error adjustment of movement dynamics (*F*_(2,136)_ = 0.87, *p* = .420, *BF_01_* = 6.85). Participants also showed comparable modification of their communicative strategies following negative feedback, with no group differences (*F*_(2,141)_ = 0.42, *p* = .656, *BF_01_* = 10.32; Supplemental Figure S3b). Taken together, these findings indicate that autistic and non-autistic participants were equally responsive to explicit feedback.

### Effects of early social exposure

Among non-autistic participants, greater daycare attendance during early childhood was associated with steeper declines in stereotype-driven adjustments (*ρ*_(73)_ = −0.22, *p_one-sided_* = .030; Figure 3(d)). This relationship remained marginally significant after controlling for familial factors such as number of siblings and socioeconomic status (*ρ*_(71)_ = −0.19, *p_one-sided_* = .051). The effect size closely matched that reported in a larger developmental sample (*ρ*_(88)_ = −0.23; (Koch et al., 2024)), with no significant difference between studies (*z* = −0.07, *p* = .474). In contrast, autistic participants showed no significant association between daycare attendance and interaction-driven adjustments (*ρ*_(40)_ = 0.24, *p_two-sided_* = .123; Figure 3(e)). The strength of these associations differed significantly between groups (*z* = −2.38, *p* = .009), despite equivalent daycare attendance across groups (*F*_(1,122)_ = 0.07, *p* = .795, BF_01_ = 4.89). When autistic and non-autistic participants were combined, no significant association was found (*ρ*_(116)_ = −0.04, *p_two-sided_* = .674), and the effect was significantly attenuated relative to the non-autistic group alone (*z* = 1.98, *p_two-sided_* = .024). Overall, these findings support prior evidence that early social experiences shape interaction-driven adjustments into adulthood but show no comparable benefits in autistic participants.

## Discussion

Our findings demonstrate that, when relying on stereotypes, autistic individuals are equally motivated and capable of adjusting their communication to meet the presumed abilities of their interaction partners. Like non-autistic peers with varying levels of social anxiety, autistic participants spontaneously emphasized communicative behaviors when interacting with a partner believed to be a child compared to one believed to be an adult. These adjustments were driven entirely by stereotypical beliefs, as both roles were performed by the same role-blind confederate exhibiting matched behaviors. However, unlike non-autistic participants, who gradually adapted their communication as accumulating evidence contradicted initial assumptions about partner differences, autistic individuals maintained their stereotype-driven adjustments throughout the interaction. Developmentally, early social exposure in daycare predicted greater sensitivity to interaction-based evidence in non-autistic participants, a relationship absent in the autism group. Together, these results illuminate both cognitive and developmental factors shaping interpersonal communication in autism and neurotypical development.

While stereotypes guided the initial approach of both autistic and non-autistic individuals, only non-autistic participants dynamically modulated their reliance on these assumptions to accommodate evidence arising from the interaction (Grice, 1957). This distinction helps reconcile discrepancies between structured task-based assessments, where autistic individuals often demonstrate proficiency in adopting a generic partner’s perspective (Begeer et al., 2010; Bowler, 1992; Pantelis & Kennedy, 2017; van Tiel et al., 2021), and dynamic conversational contexts, in which they appear less responsive to a partner’s evolving understanding (Alkire et al., 2023; Dahlgren & Dahlgren Sandberg, 2008; Nadig et al., 2015; Paul et al., 2009; Volden et al., 1997). By disentangling stereotype-driven from interaction-driven communicative adjustments in live exchanges, the present findings suggest that these contrasting outcomes reflect distinct underlying processes. Consistent with emerging evidence for separate neuroanatomical and developmental pathways (Koch et al., 2024), our results indicate that perspective-taking involves not only applying stereotype-based generalizations but also contextualizing this knowledge in real time to accommodate interactional evidence.

The results also challenge the notion of universally diminished social motivation or cognitive flexibility in autism. Autistic participants, like their non-autistic counterparts, spontaneously tailored their communicative behavior to presumed partner abilities, even when these adjustments had no measurable impact on communicative success. Moreover, they promptly adapted their behavior in response to explicit feedback signaling misunderstanding. This pattern points to a motivation for the success of the social exchange itself, rather than solely for individual task performance, aligning with evidence of spontaneous helping behaviors and self-reported social interest among many autistic individuals (Jaswal & Akhtar, 2018; Liebal et al., 2008; Morrison et al., 2020).

A more plausible explanation for the lack of dynamic adaptation lies in reduced sensitivity to implicit cues that conflicted with presumed partner differences. Across-trial consistencies embedded in the confederate’s behavior, such as characteristic interpretation patterns or confidence displayed when moving the squirrel, were not critical for trial-level outcomes but may nevertheless have led non-autistic participants to treat both partners equivalently. Autistic participants appeared less influenced by such contextual regularities, consistent with evidence of reduced sensitivity to partner-specific variations in engagement or talkativeness (Cola et al., 2022). These findings point to fundamental differences in cognitive processing (Frith, 2003; Minshew & Goldstein, 1998): whereas non-autistic individuals dynamically integrate contextual patterns unfolding across interactions (Brennan et al., 2010; Clark, 1996; Stolk et al., 2022), autistic individuals place greater weight on trial-level parameters and outcomes, reinforcing stable communicative behaviors tailored to each partner (Husain et al., 2021).

The developmental findings further refine our understanding of how diverse social contexts shape interpersonal communication. Prior work has demonstrated lasting benefits of early social exposure, such as multilingual environments enhancing perspective-taking (Liberman et al., 2017) or cross-racial experiences shaping emotion recognition (Telzer et al., 2013). Consistent with this, our study shows that early exposure to daycare environments enhances sensitivity to interaction-based evidence into adulthood. Daycare provides children with opportunities to interact with diverse peers and caregivers, fostering adaptability across varied interactive contexts. However, the absence of this developmental relationship in the autism group suggests that such benefits are not conferred automatically. Autistic children may not fully utilize opportunities for social interaction, and active engagement may be necessary to foster adaptability (Fasano et al., 2021; Stone & Caro-Martinez, 1990). A key direction for future research is to explore strategies that promote extended exchanges, such as structured interactions that encourage turn-taking, to maximize these benefits (Curcio & Paccia, 1987; Friedman et al., 2019).

A potential critique is that diminished sensitivity to interactional evidence should have hindered task performance in autistic individuals. Indeed, prior work with a similar task found reduced success when autistic participants had to align solutions under unpredictable conditions (Wadge et al., 2019). By contrast, the present paradigm used a fixed problem space in which all possible acorn locations were visible from the outset and solutions were generated individually. This design minimized alignment demands in favor of skills such as consistency and abstract spatial reasoning, areas in which autistic individuals are known to exhibit relative strengths (Lawson et al., 2017; Sevgi et al., 2020; Stevenson & Gernsbacher, 2013). A related critique might be that partner-related adjustments should have facilitated success with the presumed child partner, since they emphasized relevant areas of the game board. However, these adjustments were quantifiably small, accounting for just 5% of the time spent in the acorn’s location and were largely unnoticed by both confederates and participants. This suggests they reflected an implicit bias rather than a deliberate strategy critical for task completion.

Finally, the study revealed no significant effects of heightened social anxiety on task performance or communicative adjustments. The absence of direct gaze, physical co-presence, and turn-taking demands, combined with the comfort of participating from a familiar environment, likely reduced the salience of social pressures in this paradigm (Hutchins et al., 2021). These findings support the view that communication challenges in autism stem less from the immediate demands of face-to-face engagement, although such demands may amplify difficulties in other settings, and more from fundamental differences in how interactional evidence is processed and used to adapt to a partner’s understanding (Livingston et al., 2020; Wadge et al., 2019). The developmental results further reinforce this notion, pointing to altered influences well before the typical onset of social anxiety in autism (White et al., 2009).

In conclusion, autistic individuals demonstrated comparable motivation and ability to tailor their communication to presumed partner abilities but faced challenges in adapting to their partners’ actual understanding. These findings highlight an important societal concern: although reduced adaptation can readily be perceived as diminished social interest, the substantial and sustained effort many autistic individuals invest in social engagement at the same time suggests that such an interpretation may misrepresent their social motivation and lead to unintended social consequences.

## Supplemental Material

sj-docx-1-aut-10.1177_13623613251410418 – Supplemental material for Partner-dependent communication without dynamic adaptation in autism Supplemental material, sj-docx-1-aut-10.1177_13623613251410418 for Partner-dependent communication without dynamic adaptation in autism by Saskia BJ Koch, Jordy van Langen, Jana Bašnáková and Arjen Stolk in Autism

sj-pdf-2-aut-10.1177_13623613251410418 – Supplemental material for Partner-dependent communication without dynamic adaptation in autism Supplemental material, sj-pdf-2-aut-10.1177_13623613251410418 for Partner-dependent communication without dynamic adaptation in autism by Saskia BJ Koch, Jordy van Langen, Jana Bašnáková and Arjen Stolk in Autism
