# Supplementary material for: Partner-dependent communication without dynamic adaptation in autism
Source: Autism. 2026 Jan 31;30(3):736–47. doi: 10.1177/13623613251410418 (PMC12923631; doi:10.1177/13623613251410418)
Supplement: sj-docx-1-aut-10.1177_13623613251410418 – Supplemental material for Partner-dependent communication without dynamic adaptation in autism [file sj-docx-1-aut-10.1177_13623613251410418.docx]

**SUPPLEMENTAL RESULTS**

**Partner Manipulation**

Following task performance, 133 participants (92.36%) completed an online questionnaire assessing their perceptions of the communicative partners they had interacted with in the game [(de Boer et al. 2017; Newman-Norlund et al. 2009)](https://paperpile.com/c/3mZd8V/m55vJ+vfXOM). This allowed evaluation of whether participants understood and believed the manipulation of differing partner capabilities.

***Perceived age and similarity.*** When asked to estimate the age of their partners, participants identified the presumed child partner as younger (7.41 ± 4.68 years) than the presumed adult partner (25.92 ± 7.05 years). A repeated-measures ANOVA confirmed a significant difference in estimated age between the partners (partner: *F*_(1,124)_ = 719.93, *p* < .001, *η^2^g* = .709), independent of group status (group: *F*_(2,124)_ = .918, *p* = .402, *η^2^g* = .009; group-by-partner interaction: *F*_(2,124)_ = 1.14, *p* = .322, *η^2^g* = .008). Participants also rated the adult partner (5.38 ± 1.67) as more similar to themselves than the child partner (4.66 ± 2.00) on a scale from 1 (nothing like you) to 10 (exactly like you). This difference was significant (partner: *F*_(1,130)_ = 12.15, *p* < .001, *η^2^* = .037) but did not vary by group (group: *F*_(2,130)_ = 0.80, *p* = .454, *η^2^* = .007; group-by-partner interaction: *F*_(2,130)_ = 0.16, *p* = .854, *η^2^ =* .001).

***Perceived cognitive abilities.*** Participants assessed the cognitive abilities of the presumed child and adult partners across nine domains (yes/no), shown in Figure S2. The proportion of participants attributing each ability was compared to chance (50%) using exact binomial tests, and group differences were tested with chi-square analyses. Bonferroni correction was applied for 18 comparisons (*p* < .0027). Most participants believed both partners could ride a bike (child: 94.74%, *p* < .001; adult: 100%, *p* < .001), sing the Dutch children’s song ‘zakdoekje leggen’ (child: 94.74%, *p* < .001; adult: 91.67%, *p* < .001), and count to 100 (child: 69.17%, *p* < .001; adult: 98.48%, *p* < .001). However, counting to 100 was attributed significantly more often to the adult partner (two-sample sign test: *p* < .001). Participants overwhelmingly believed only the adult partner could solve multiplication problems (child: 41.67%, *p* = .067; adult: 98.48%, *p* < .001), drive a car (child: 6.01%, *p* <.001; adult: 91.67%, *p* < .001), go to the store alone (child: 25.56%, *p* < .001; adult: 97.73%, *p* < .001), and read *The Diary of Anne Frank* (child: 16.54%, *p* < .001; adult: 95.45%, *p* < .001). Proportions significantly differed between partners (all *p* < .001). On the other hand, participants believed only the child partner was likely to have stuffed animals (child: 93.18%, *p* <.001; adult: 51.51%, *p* = 0.794) and plays video games (child: 84.85%, *p* < .001; adult: 59.09%, *p* = .045), which these abilities significantly more often attributed to the child partner (all *p* < .001). No significant group differences were observed in the assessment of the partners’ perceived cognitive abilities (all *p* > .27).

***Behavioral and performance perceptions.*** A minority of participants (14.29%) reported intentionally slowing their movements when interacting with the child partner. This proportion did not differ significantly between groups (𝜒²_(2)_ = 0.89, *p* = .642, *BF_01_* = 22.67), indicating that most participants were not consciously aware of adjusting their behavior to their partners’ presumed abilities. When asked which partner performed better in the communication game, participants showed no clear preference (child better: 40.60%, adult better: 32.33%, equal performance: 27.07%), with no significant group differences (𝜒²_(4)_ = 2.25, *p* = .691, *BF_01_* = 44.87).

***Outliers.*** Among the 133 participants who completed the online questionnaire, 17 (12.8%) expressed doubts about the partner manipulation in the comments section, with these doubts being independent of group status (𝜒²_(2)_ = 0.88, *p* = .645, *BF_01_* = 24.53). Additionally, one participant incorrectly perceived the adult partner as elderly and less capable than the child partner. To ensure the validity of our analyses, participants who did not understand or believe the manipulation were excluded, particularly if their average stereotype-driven adjustment was negative, indicating a failure to adapt their behavior toward the younger, presumed less capable child partner. This exclusion resulted in the removal of five participants (~3% of 148 total), including two from the autism group and three from the low social anxiety group.

Taken together, these results highlight clear distinctions in participants’ perceived cognitive abilities between the child and adult partners. Notably, these beliefs were consistent across participant groups.

**Confederate Performance**

We conducted additional analyses to confirm that the confederate experimenter exhibited consistent behavior across the roles of child and adult partner, focusing on planning time, movement time, and comprehension.

***Planning and movement time.*** Repeated-measures ANOVAs were conducted on the confederate’s planning time and movement time, with partner role (child, adult) as the within-subjects factor and group (autism, low SA, high SA) as the between-subjects factor (R’s *rstatix* package). Both planning time and movement time were log-transformed to meet normal distribution requirements, and one outlier for movement time was excluded. A significance threshold of *p* < .017 was applied to correct for multiple comparisons (*p* < .05/3 dependent variables, i.e. planning time, movement time, and comprehension). The analyses showed no significant differences in the confederate’s planning time (*F_(1,136)_* = 2.14, *p* = .145, *BF_01_* = 3.06) or movement time (*F_(1,135)_* = 0.22, *p* = .639, *BF_01_* = 6.80) between roles. Additionally, no significant effects of group status were observed in the confederate’s planning time (group: *F_(2,136)_* = 0.64, *p* = .531, *BF_01_* = 5.12; group-by-partner interaction: *F_(2,136)_* = 1.60, *p* = .205, *BF_01_* = 3.81) or movement time (group: *F_(2,135)_* = 0.47, *p* = .624, *BF_01_* = 4.61; group-by-partner interaction: *F_(2,135)_* = 0.76, *p* = .470, *BF_01_* = 7.60).

***Comprehension.*** The confederate’s comprehension was assessed using Bayesian linear mixed models implemented in R’s *brms* package [(Bürkner 2017)](https://paperpile.com/c/3mZd8V/kjAGJ). These models used a Bernoulli distribution (logit link) with communicative success (0, 1) as the dependent variable, partner role (child, adult) as the within-subjects factor, group (autism low SA, high SA) as the between-subjects factor, task difficulty as covariate, and a random intercept for each participant. The results revealed no significant difference in comprehension between the child and adult roles (*B* = -0.12, 95% CI = [-0.30 0.06], *pp* = .192). Furthermore, no significant interaction effects of group status were found (partner-by-group interaction for low SA vs. autism: *B* = 0.16, 95% CI = [-0.09 0.41], *pp* = .20; partner-by-group interaction for high SA vs. autism: *B* = 0.07, 95% CI = [-0.18 0.31], *pp* = .512).

These results indicate that the confederate maintained consistent behavior across the child and adult partner roles. This consistency confirms that any behavioral adjustments by participants were driven solely by their beliefs about their partners’ differing cognitive abilities.

**Communicative Specificity**

We conducted several control analyses to confirm that participants’ partner-related adjustments were specific to communicatively relevant areas of the game board and did not influence other aspects of their behavior, including participants’ planning time, movement time, number of moves, and time spent on unrelated locations. Separate repeated-measures ANOVAs were performed for each dependent variable, with presumed partner (adult, child) as the within-subjects factor and group (autism, low SA, high SA) as the between-subjects factor. All dependent variables were log-transformed to meet normal distribution assumptions, and a Bonferroni correction was applied to account for multiple comparisons, with a significant threshold of *p* < .013 (0.05/4 dependent variables).

***Planning time.*** Participants’ planning time did not differ between presumed partners (*F*_(1,136)_ = 0.04, *p* = .842, *BF_01_* = 7.35). There were no significant effects of group (*F*_(2,136)_ = 1.63, *p* = .199, *BF_01_* = 1.54) or group-by-partner interactions (*F*_(2,136)_ = 1.07, *p* = .344, *BF_01_* = 5.16).

***Movement time.*** Participants’ movement time, encompassing time spent on the acorn’s location and other areas of the game board, differed significantly between partners (*F_(1,136)_* = 25.21, *p* < .001). Notably, this effect was primarily driven by partner-related variations in time on the acorn’s location, reflecting stereotype-driven adjustments, while time spent on other locations of the board remained consistent across partners, as shown below. Importantly, movement time was not affected by group status (*F_(2,136)_* = 0.30, *p* = 0.745, *BF_01_* = 1.86), nor were there significant group-by-partner interactions (*F*_(2,136)_ = 0.85, *p* = .429, *BF_01_* = 6.69).

***Number of moves.*** The number of moves was not significantly influenced by the presumed partner (*F*_(1,135)_ = 1.55, *p* = .215, *BF_01_* = 2.33). Additionally, no significant effects of group (*F*_(2,135)_ = 0.15, *p* = .858, *BF_01_* = 2.06) or group-by-partner interactions (*F*_(2,135)_ = 0.18, *p* = .832, *BF_01_* = 13.17) were observed. Note, one outlier was removed.

***Time spent on other locations.*** Time spent on other game board locations was unaffected by the presumed partner *F*_(1,136)_ = 1.29, *p* = .259, *BF_01_* = 4.20). Similarly, no significant effects of group (*F*_(2,136)_ = 0.44, *p* = .647, *BF_01_* = 2.37) or group-by-partner interactions (*F*_(2,136)_ = 0.64, *p* = .529, *BF_01_* = 7.98) were found (Figure S1).

These results demonstrate that participants’ partner-related adjustments were specific to communicatively relevant aspects of their behavior and were comparable across autistic and non-autistic participants.

**Communicative Strategies**

We conducted several control analyses to test whether autistic and non-autistic participants generated comparable communicative strategies. Strategies were manually annotated by a research assistant blind to participants’ group status, following a classification system previously developed for this communication game [(de Boer et al. 2017)](https://paperpile.com/c/3mZd8V/m55vJ), see Table S2. First, we calculated the number of distinct strategies each participant used across the game. Group differences in strategy variety were assessed via a one-way ANOVA with group (autism, low SA, high SA) as the between-subjects factor. Second, we compared the frequency distributions of strategies between groups using Kullback-Leibler (KL) divergence. Finally, we examined whether autistic and non-autistic participants were equally likely to modify their communicative strategy in the trial following a misunderstanding. The percentage of strategy modifications in error trials was compared with a one-way ANOVA with group as the between-subjects factor.

Autistic (4.10 ± 0.93), low SA (4.12 ± 1.10), and high SA participants (4.27 ± 0.87) employed a comparable number of distinct strategies during the communicative interaction (*F*_(2,141)_ = 0.42, *p* = .659, *BF_01_* = 10.29). The distributions of strategy frequencies were also closely matched across groups (low SA vs. autism: KL divergence = 0.075; high SA vs. autism: KL divergence = 0.072; low SA vs. high SA: KL divergence = 0.061; Figure S3a). Finally, autistic and non-autistic participants were equally likely to modify their strategy following a misunderstanding (*F*_(2,141)_ = 0.42, *p* = .656, *BF_01_* = 10.32; Figure S3b).

Taken together, these results indicate that autistic and non-autistic participants generated comparable sets of communicative strategies and were equally inclined to adjust their strategies after a misunderstanding.

**Table S1. Demographic data**

|  | *Group* | | |  | *Group differences* | |
| --- | --- | --- | --- | --- | --- | --- |
|  | *Autism (n = 48)* | *Low SA (n = 48)* | *High SA (n = 48)* |  | *Test statistic* | *Bayes Factor* |
| Age (years) | 27.77 (6.47) | 26.58 (5.44) | 26.25 (5.26) |  | *F_(2,141)_* = 0.93 | BF_01_ = 6.70 |
| Sex (females) | 28 (58.33%) | 25 (52.08%) | 30 (62.50%) |  | 𝜒²_(2)_ = 1.08 | BF_01_ = 11.09 |
| Verbal IQ (WAIS-III)^1^ | 123.11 (15.34) | 128.40 (12.98) | 124.62 (15.42) |  | *F_(2,140)_* = 1.65 | BF_01_ = 3.62 |
| Nonverbal IQ (RPM)^2^ | 104.21 (9.27) | 102.13 (12.90) | 101.69 (11.68) |  | *F_(2,140)_* = 0.67 | BF_01_ = 8.27 |
| Autism-Spectrum  Quotient (AQ-50)^2^ | 30.98 (7.57) | 13.06 (5.46) | 19.65 (6.96) |  | *F_(2,140)_* = 86.26** | BF_10_ > 100 |
| Social anxiety (LSAS) | 53.54 (25.22) | 14.58 (7.65) | 53.12 (18.83) |  | *F*_(2,75.72)_ = 124.47** | BF_10_ > 100 |
| Daycare (days/week)^3^ | 1.12 (0.84) | 0.99 (0.92) | 1.33 (1.20) |  | *F*_(2,121)_ = 1.20 | BF_01_ = 4.81 |

*Values are given as frequency (percentage) for categorical variables and mean (standard deviation) for continuous variables. WAIS-III, Wechsler Adult Intelligence Scale* [*(Wechsler 1997)*](https://paperpile.com/c/3mZd8V/2xVRy)*; RPM, Raven’s Progressive Matrices* [*(Raven 1989)*](https://paperpile.com/c/3mZd8V/xih4G)*; BF_01_, Bayes Factor in favor of the null hypothesis; BF_10_, Bayes Factor in favor of the alternative hypothesis of group differences; 1, verbal IQ missing for 1 autistic participant; 2, nonverbal IQ and AQ missing for 1 low SA participant; 3, daycare data missing for 3 autistic, 10 low SA and 7 high SA participants; **, p < .001*

**Table S2. Strategy classification**

| *Strategy* | *Description* |
| --- | --- |
| Target only | The bird is kept longer in the square containing the acorn than in other visited squares on the game board. |
| Target entry/exit | The bird enters or leaves the square containing the acorn through the side nearest to the acorn’s circle, indicating its precise location within the square. |
| Target anchor | The bird steps out of and back into the square containing the acorn via the side directly adjacent to the acorn’s circle, thereby anchoring its precise location. |
| Adjacent anchor | As in the Target anchor, but the stepping in and out occurs toward a square adjacent to the acorn’s square, not toward the acorn’s circle. |
| Center anchor | As in the Target anchor, but the stepping in and out occurs from the central square, oriented toward the acorn’s circle, either before or after visiting the acorn’s square. |
| Draw | The bird is moved across multiple squares to depict the spatial configuration of the acorn’s circle within its square. |
| Circle | The bird is moved in a circular path across multiple squares, thereby indicating the orientation of the acorn’s circle. |
| Line | The bird is moved in a straight line across squares, aligned with the acorn’s circle. |

**
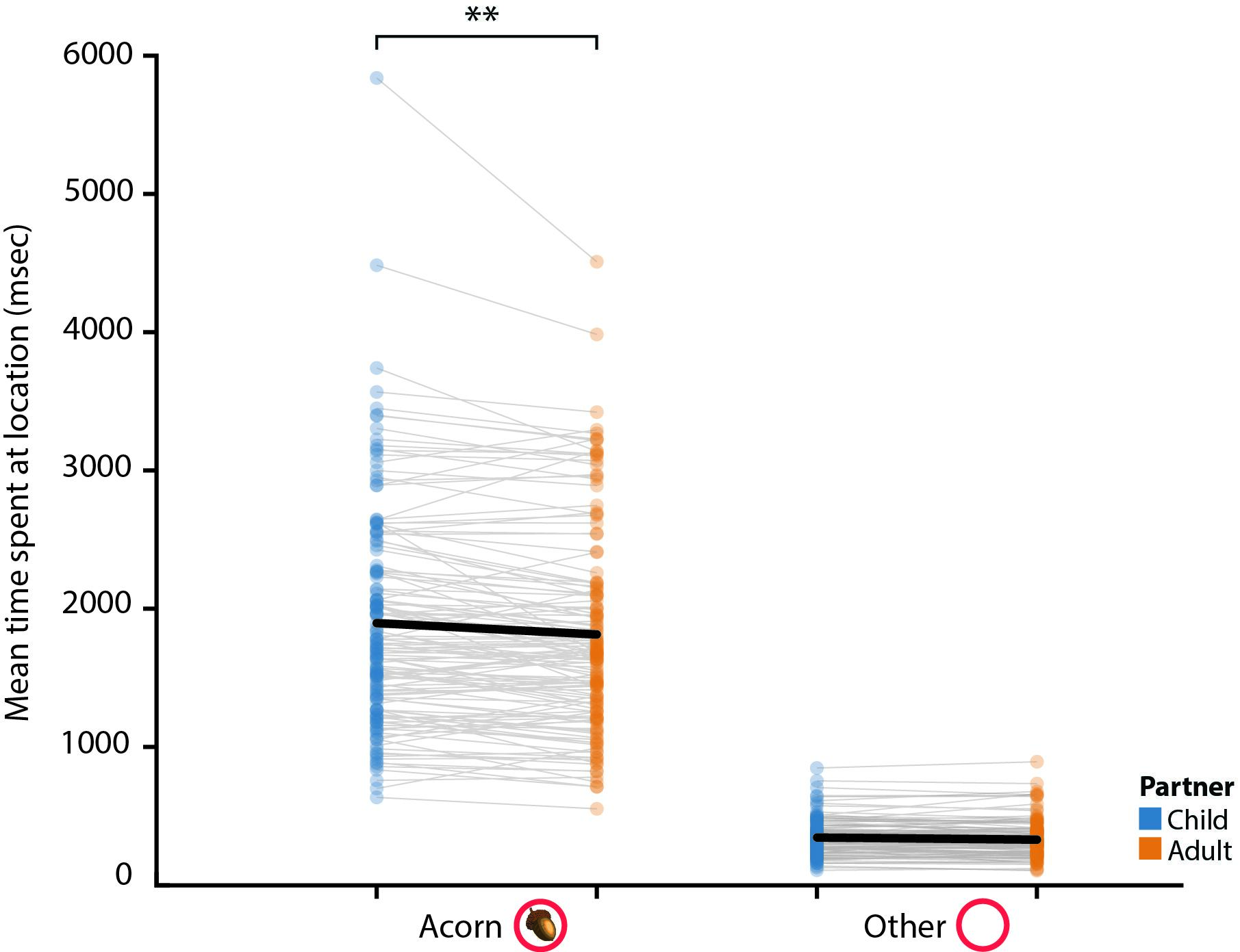
**

**Figure S1. Time spent in different game board locations for the presumed child and adult partners** Participants spent significantly more time with the bird avatar in the grid square containing the acorn when communicating with the presumed child partner compared to the presumed adult partner (*M difference* = 94.48 ms, *F*_(1,136)_ = 24.02, *p* < .001). In contrast, no significant difference was observed for the time spent in other locations of the board (*M difference* = 3.06 ms, *F_(1,136)_* = 1.29, *p* = .259, *BF_01_* = 4.20), indicating that the partner-related movement adjustments were specific to communicatively relevant information. Individual data points are represented as dots, with bold black lines indicating sample averages; ** *p* < .001.


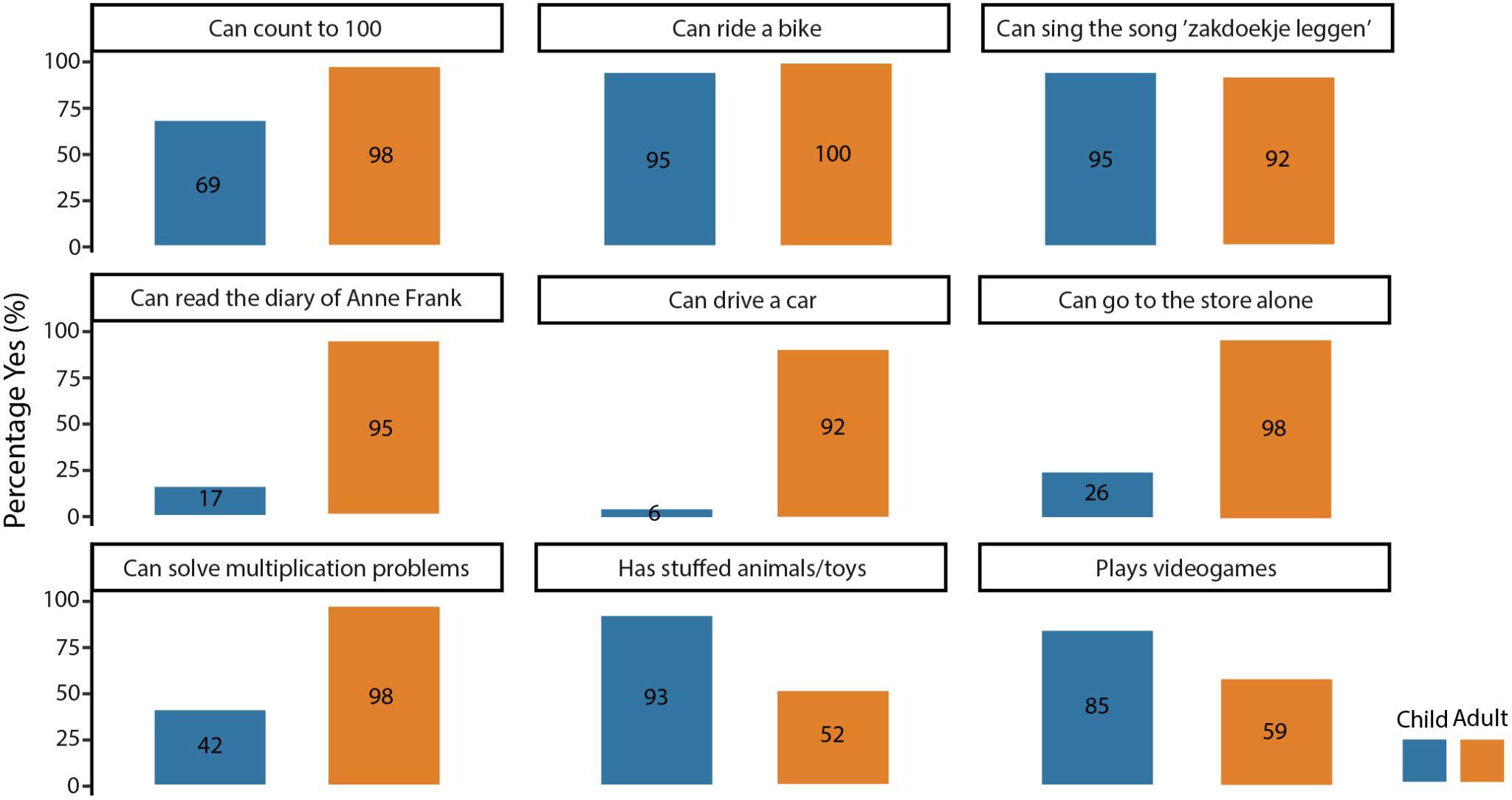


**Figure S2. Perceived cognitive abilities of the presumed child and adult partners**

Bar charts illustrating the proportion of participants attributing specific cognitive abilities to the presumed child and adult partners across nine domains. Most participants believed both partners could count to 100, ride a bike, and sing a Dutch children’s song. Abilities such as reading *The Diary of Anne Frank*, driving a car, going to the store alone, and solving multiplication problems were overwhelmingly attributed to the adult partner. Conversely, owning stuffed animals and playing video games were attributed significantly more often to the child partner.


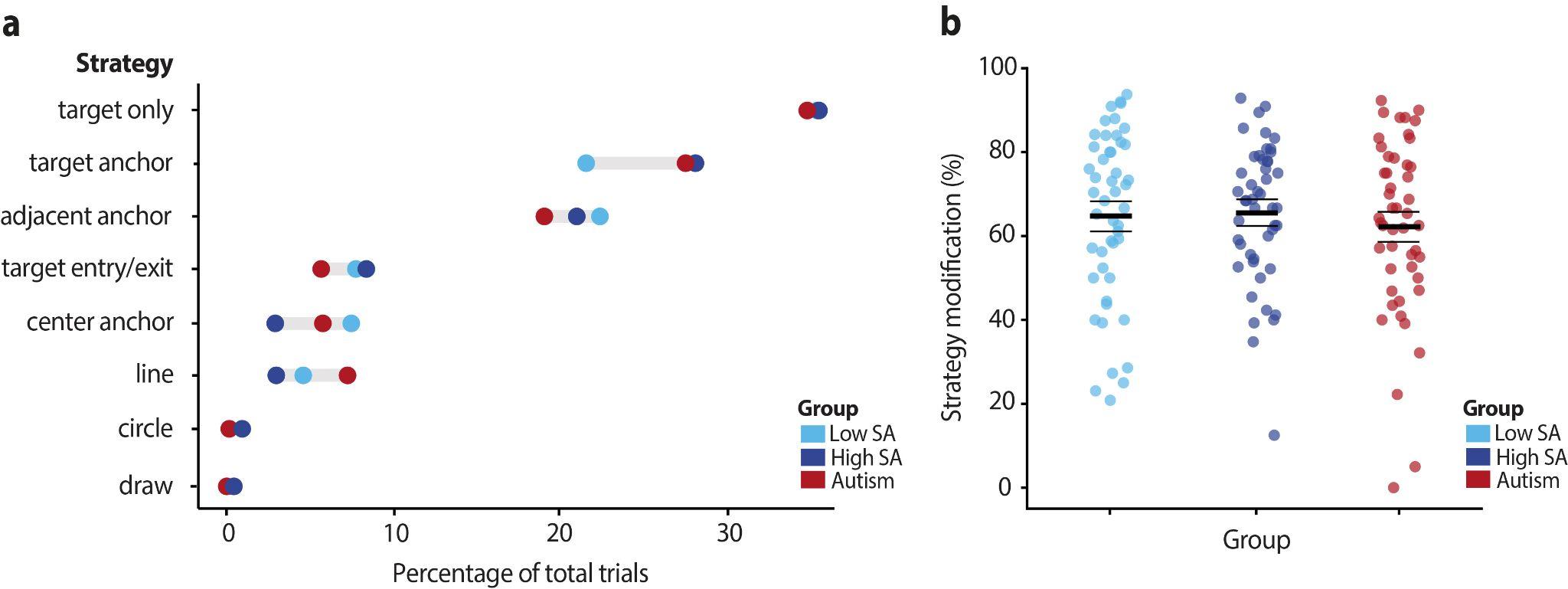


**Figure S3. Comparable strategy use and modification across groups.** (**a**) Autistic and non-autistic participants employed a similar variety and distribution of communicative strategies during the game. See Table S2 for descriptions of the strategies. (**b**) Participant groups were equally likely to modify their communicative strategies following a misunderstanding (percentage of total error trials). SA, social anxiety.
